# HIV-Prevalence in South Africa by settlement type: A repeat population-based cross-sectional analysis of men and women

**DOI:** 10.1371/journal.pone.0230105

**Published:** 2020-03-17

**Authors:** Andrew Gibbs, Tarylee Reddy, Kristin Dunkle, Rachel Jewkes

**Affiliations:** 1 Gender and Health Research Unit, South African Medical Research Council, Pretoria, South Africa; 2 Centre for Rural Health, School of Nursing and Public Health, University of KwaZulu-Natal, Durban, South Africa; 3 Biostatistics Unit, South African Medical Research Council, Durban, South Africa; 4 Office of the Executive Scientist, South African Medical Research Council, Pretoria, South Africa; 5 School of Public Health, University of Witwatersrand, Johannesburg, South Africa; University of Lleida, SPAIN

## Abstract

To assess i) whether there is an independent association between HIV-prevalence and settlement types (urban formal, urban informal, rural formal, rural informal), and, ii) whether this changes over time, in South Africa. We draw on four (2002; 2005; 2008; 2012) cross-sectional South African household surveys. Data is analysed by sex (male/female), and for women by age categories (15–49; and 15–24; 25–49) at all-time points, for men in 2012 data is analysed by age categories (15–24; 25–49). By settlement type and sex/age combinations, we descriptively assess the association between socio-demographic and HIV-risk factors; HIV-prevalence; and trends in HIV-prevalence by time. Relative risk ratios assess unadjusted and adjusted risk for HIV-prevalence by settlement type. All estimates are weighted, and account for survey design. In all survey years, and combinations of sex/age categorisations, HIV-prevalence is highest in urban informal settlements. For men (15–49) an increasing HIV-prevalence over time in rural informal settlements was seen (p = 0.001). For women (15–49) HIV-prevalence increases over time for urban informal, rural informal, rural formal, and women (15–24) decreases in urban formal and urban informal, and women (25–49) increases urban informal and rural informal settlements. In analyses adjusting for potential socio-demographic and risk factors, compared to urban formal settlements, urban informal settlements had consistently higher relative risk of HIV for women, in all age categorisations, for instance in 2012 this was RR1.89 (1.50, 2.40) for all women (15–49), for 15–24 (RR1.79, 1.17–2.73), and women 25–49 (RR1.91, 1.47–2.48). For men, in the overall age categorization, urban informal settlements had a higher relative risk for HIV in all years. In 2012, when this was disaggregated by age, for men 15–24 rural informal (IRR2.69, 1.28–5.67), and rural formal (RR3.59, 1.49–8.64), and for men 25–49 it was urban informal settlements with the highest (RR1.68, 1.11–2.54). In 2012, rural informal settlements also had higher adjusted relative risk for HIV-prevalence for men (15–49) and women (15–49; 15–24; 25–49). In South Africa, HIV-prevalence is patterned geographically, with urban informal settlements having a particularly high burden. Geographical targeting of responses is critical for the HIV-response.

## Introduction

HIV-incidence and HIV-prevalence is spatially distributed globally, nationally, and sub-nationally. The spatial patterning of HIV across settings reflects inequalities in access to resources, healthcare services, and power differentials, particularly along lines of poverty, sexuality, gender and race [1–3]. Across Africa (excluding South Africa) a limited body of work has looked at this by settlement type. In Kenya, HIV-prevalence was assessed comparing urban slums, with urban-non slum settlements, with the HIV-prevalence 12% and 5% respectively [4]. While in Namibia’s capital city, Windhoek, hotspot mapping identified particularly high HIV-incidence in informal settlement areas [5]. Understanding the spatial patterning of HIV-prevalence globally, and nationally, is critical to ensure limited resources are targeted most effectively, particularly as donor funding is declining [6].

In South Africa, a number of studies have explored the spatial patterning of HIV. Studies have, for instance, sought to highlight HIV ‘hot-spots’. For example, Tanser et al [7] found wide variation in HIV-prevalence (from 6–39%) in a rural demographic health surveillance area in KwaZulu-Natal, with clustering of HIV in homesteads [8], nearer national roads [7], and by transport corridors [9]. Amongst women screened for enrollment in clinical trials, also in KwaZulu-Natal, but in more urban settings, HIV-prevalence clustered in ‘hot spots’ with HIV-prevalence in those ranging from 56.0% to 39.0%; with distinguishing factors including being in peri-urban communities (typically including informal settlements), and reporting more lifetime sexual partners [10]. Finally, one nationally representative youth (15–24) survey, found that HIV-prevalence was pattern by rural/urban status, with higher HIV-prevalence in urban settings, and was highest for young women in urban informal settlements (but not men) [11].

In addition, in South Africa, there have been five nationally representative population based studies of HIV-prevalence and incidence, in 2002, 2005, 2008, 2012, and 2017 [12–16]. In each of these data on settlement type is collected and analysed in the main report. The 2017 data is not yet publicly available, and in the report that has been produced, settlement types have been coded differently to previous studies, combing urban informal and urban formal to create one category of ‘urban’. All four previous (2002; 2005; 2008; 2012) nationally representative studies analysed data by urban formal, urban informal, rural formal and rural informal, and these analyses have described HIV-prevalence being substantially higher in urban informal settlements, compared to other settlement types [13–15].

However these nationally representative data have not be disaggregated settlement type by age and sex, which are important factors in the patterning of the HIV-epidemic, with younger (15–24) women at greater risk of acquiring HIV than younger men, and women of all ages more likely to be living with HIV, compared to men [17]. Additionally, the prior descriptive work provided only prevalence estimates, without adjustment for the distribution of potential socio-demographic differences that might account these spatial differences. Nor have changes in HIV-prevalence and settlement-type been explored by time.

Currently, South Africa spends US$1.9 billion annually on HIV, with a quarter (U$0.5 billion) coming from international donors [18]. And while there are a number of large-scale HIV-prevention, and treatment programmes, including universal test-and-treat, and the Determined, Resilient, Empowered, AIDS-Free, Mentored, and Safe (DREAMS) programme for young women (15–24) [19], donors are reducing their funding [18]. Understanding the HIV-epidemic in more detail, by sex and age, will help focus targeting of limited resources.

Drawing on representative population-based data in South Africa of four surveys (2002; 2005; 2008 and 2012), this paper has three objectives. First, to describe HIV-risk acquisition factors by settlement type and age and sex. Second, to describe HIV-prevalence by settlement-type and assess trends over time by settlement-type. Third, to assess whether settlement-type independently predicts HIV-prevalence after accounting for risk factors.

## Materials and methods

### Data collection

We used four nationally representative population-based cross-sectional household surveys from 2002, 2005, 2008 and 2012 in South Africa. The National Prevalence Surveys employed a stratified multistage cluster sampling approach. In the first stage 1000 census enumeration areas (EAs) were selected proportional to size and stratified by province, geotype(settlement type) and race; after which a fixed number of households were selected per EA in the second stage. Sampling weights were calculated to correct for unequal probabilities of selection and non-response at the EA, household and individual level. The individual weights for each survey were benchmarked to relevant mid-year population estimates by age, race, sex, and province. This ensured that the sample for each survey was representative of the population distribution in South Africa for sex, age, race, settlement type, and province. Response rates for HIV-testing varied, with lower response rates consistently by men, compared to women (e.g. in 2012, for those older than 15, the testing response rate for females was 57.7%, compared to 42.3% in males) [14]. Detailed information about survey design, sampling methods, refusal rates, and survey administration is available in individual reports [12–15]. The comparability of design makes these four surveys nationally representative data sets, enabling assessment of longitudinal change across them.

All studies received ethical approval from the Human and Social Research Council’s (HSRC) ethics committee before starting, and participants provided informed consent for questionnaires, and separately for HIV-testing. Information on this can be found in the original reports.

### Outcome

The primary outcome for analysis is a binary HIV-status (positive or negative). Testing strategy varied by data collection year. In 2002 and 2005 oral saliva samples were obtained and tested using ELISA kits, with confirmatory testing on positive tests only in 2005 [12, 15]. The sensitivity and specificity of the ELISA test are 99% and 99% respectively [12, 15]. In 2008 and 2012, dried blood spots were collected and tested, and confirmatory tests conducted on all positive samples (and a 10% sub-set of negative samples) using a combination of three HIV-1 enzyme immunoassays, with 99% specificity and sensitivity [13, 14].

Classification of households into settlement type was done through coding of Enumeration Areas (EA) using census data and classifications provided by Statistics South Africa. Four options were available: urban formal, urban informal, rural formal (including commercial farms), and rural informal, which included tribal authority areas [12–15]. According to Statistics South Africa, urban areas are continuously built-up areas, and either urban formal–defined as land proclaimed as residential where settlements are structured and organized. In addition, services are provided through local government and development is controlled. Or, urban informal, which are unplanned settlements on land not defined as residential, and comprising of informal dwellings. Rural areas are any area not deemed as urban, and comprises of rural informal, which areas are controlled by tribal authorities and informal settlements outside of urban areas. In earlier surveys this was just termed tribal authority areas. And rural formal settlements, which are formal settlements outside of urban areas, which do not fall under tribal authority areas, including commercial farms [20].

### Covariates

In addition to age and sex, we identified comparable measures across all four surveys as potential co-variates, based on known risk factors for HIV-acquisition. All studies assessed current work status [employed (formal or informal), unemployed, or student]. Education level was assessed as either: none through to secondary not completed, else completed secondary or higher. For people reporting ever having had sex, age of sexual debut, and number of past year sexual partners were assessed.

### Analysis

Analyses were done separately for men and women. For men, because of high refusal rates of HIV-testing and small sample sizes in the 2002, 2005 and 2008 surveys (for instance 31% of men refused to be tested for HIV in 2008) only one age categorization (15–49) was possible. In 2012, the larger sample size enabled age stratification by adolescents (15–24), and adult men (25–49), as well as overall (15–49). For women, we analysed all respondents (aged 15–49), and then stratified by adolescents (15–24), and adult women (25–49).

We first calculated the distribution of weighted proportions of socio-demographic and HIV-acquisition risk factors by settlement type, sex, age, and survey wave, presenting percentages and 95 percent confidence intervals (95%CI). We used chi-square tests to assess variation. We then calculated weighted HIV-prevalence by settlement type for each survey by sex, and age groups. Differences are assessed through chi-squared tests. We also assessed trends in HIV-prevalence by settlement type using chi-square tests.

To assess the potential impact of settlement type on prevalent HIV infection, we used individual level poisson regression reporting relative risk with robust standard errors, taking into account sampling weights, stratification by province, and clustering within EA. We then adjusted for education, employment status, age at first sex, number of partners in past year. We report both the unadjusted and adjusted relative risk ratios. All analyses were performed in STATA15, using the survey commands, and weighted estimations.

## Results

### Men aged 15–49

In total, 2430, 5047, 4291, and 8510 men participated in 2002, 2005, 2008 and 2012 surveys, respectively. Table 1 presents socio-demographic and HIV-acquisition risk factors for all four surveys for men aged 15–49. In 2002, a greater proportion of young men (15–24) were in rural informal settlements (58.0%), compared to other settlement types (37.4% urban formal; 37.2% urban informal; 27.6% urban formal; p<0.0001). In urban informal (43.2%) and rural informal (46.1%) settlements a significantly greater proportion reported being unemployed, compared to urban formal (24.0%) and rural formal (17.3%). The majority of men in urban informal, rural informal and rural formal had not completed secondary school (83.9%, 86.1%, 85.0% respectively), and this was significantly higher compared to urban formal (42.4%). A significantly greater proportion of men in urban informal settlements (58.1%) and rural formal (54.4%) reported sexual debut ≤18 years, compared to urban formal (47.4%), and rural informal (44.8%). In all other survey years (2005; 2008; 2012), a similar patterning of socio-demographic and sexual behaviours was seen, with little variation between years.

**Table 1:** Men 15–49 socio-demographic and risk factors by settlement types for all survey years.

**Table 1a: 2002 Survey: Men (15–49), socio-demographic and risk factors, by settlement type**											
			Urban Formal		Urban Informal		Rural Informal		Rural Formal		
	N	n	Col %	95% CI	Col %	95% CI	Col %	95% CI	Col %	95% CI	p-value
Age	2430										
15–24		1145	37.4	[33.4,41.6]	37.2	[29.2,46.1]	58.0	[51.2,64.5]	27.6	[18.8,38.5]	<0.0001
25–49		1285	62.6	[58.4,66.6]	62.8	[53.9,70.8]	42.0	[35.5,48.8]	72.4	[61.5,81.2]	
Employment status	2314										
Employed		1064	52.7	[47.6,57.7]	40.1	[31.6,49.2]	18.1	[13.9,23.4]	79.1	[66.7,87.7]	<0.0001
Unemployed		666	24.0	[20.0,28.4]	43.2	[34.5,52.5]	46.1	[39.9,52.3]	17.3	[9.9,28.6]	
Student		584	23.4	[19.9,27.2]	16.6	[11.1,24.1]	35.8	[29.9,42.2]	3.6	[1.4,8.8]	
Education level	2424										
Secondary not complete or less		1692	57.6	[51.8,63.2]	83.9	[76.9,89.1]	86.1	[81.1,89.9]	85.0	[70.2,93.2]	<0.0001
Secondary complete/higher		732	42.4	[36.8,48.2]	16.1	[10.9,23.1]	13.9	[10.1,18.9]	15.0	[6.8,29.8]	
Past year sex partners	2415										
Never had sex, none, one		2164	91.3	[88.7,93.4]	76.9	[68.0,83.9]	86.2	[80.0,90.7]	85.6	[54.4,96.7]	0.179
Two or more		251	8.7	[6.6,11.3]	23.1	[16.1,32.0]	13.8	[9.3,20.1]	14.4	[3.3,45.5]	
Age sexual debut	2396										
< = 18		1129	47.4	[42.4,52.4]	58.1	[48.3,67.3]	44.8	[38.1,51.8]	54.4	[39.7,68.5]	0.0007
> = 19		734	35.9	[31.9,40.2]	27.0	[18.6,37.4]	25.8	[20.3,32.2]	34.6	[22.7,48.8]	
Never had sex		533	16.7	[13.9,19.9]	14.9	[10.0,21.6]	29.3	[24.0,35.3]	11.0	[6.0,19.3]	
**Table 1b: 2005 Survey: Men (15–49), socio-demographic and risk factors, by settlement type**											
Age	5047										
15–24		2537	38.8	[35.8,42.0]	36.8	[30.5,43.6]	58.7	[54.3,62.9]	32.2	[23.8,41.8]	<0.0001
25–49		2510	61.2	[58.0,64.2]	63.2	[56.4,69.5]	41.3	[37.1,45.7]	67.8	[58.2,76.2]	
Employment status	4874										
Employed		2143	51.7	[47.2,56.1]	37.6	[32.2,43.3]	18.6	[15.1,22.6]	75	[66.2,82.2]	<0.0001
Unemployed		1324	26.3	[22.9,30.1]	40.7	[34.1,47.6]	38.8	[34.4,43.5]	13	[8.7,19.1]	
Student		1407	22	[19.4,25.0]	21.7	[16.7,27.8]	42.6	[38.3,47.0]	11.9	[7.3,18.9]	
Education level	4962										
Secondary not complete or less		3168	53.1	[48.3,57.9]	80.6	[75.1,85.2]	78.4	[74.0,82.3]	87.4	[80.6,92.1]	<0.0001
Secondary complete/higher		1794	46.9	[42.1,51.7]	19.4	[14.8,24.9]	21.6	[17.8,26.0]	12.6	[7.9,19.4]	
Past year sex partners	4732										
Never had sex, none, one		4248	87.1	[83.9,89.7]	84.6	[79.5,88.6]	88.6	[85.8,90.9]	92.3	[88.4,95.0]	<0.0001
Two or more		484	12.9	[10.3,16.1]	15.4	[11.4,20.5]	11.4	[9.1,14.2]	7.7	[5.0,11.7]	
Age sexual debut	4400										
< = 18		2008	50.6	[46.9,54.4]	58.3	[52.3,64.1]	41.6	[36.7,46.6]	59.3	[46.1,71.2]	0.0001
> = 19		1211	29.7	[26.4,33.2]	24.3	[19.9,29.4]	26.7	[22.8,31.0]	26.3	[18.4,36.0]	
Never had sex		1181	19.7	[17.3,22.3]	17.4	[13.1,22.7]	31.7	[27.6,36.2]	14.4	[9.2,22.1]	
**Table 1c: 2008 Survey: Men (15–49), socio-demographic and risk factors, by settlement type**											
Age	4291										
15–24		2115	40.5	[37.5,43.5]	34.1	[29.4,39.0]	56.1	[51.5,60.6]	30.4	[23.1,38.9]	<0.0001
25–49		2176	59.5	[56.5,62.5]	65.9	[61.0,70.6]	43.9	[39.4,48.5]	69.6	[61.1,76.9]	
Employment status	3999										
Employed		1893	51.7	[47.8,55.5]	48.8	[42.3,55.4]	23.1	[19.1,27.6]	78	[71.1,83.6]	<0.0001
Unemployed		959	24.1	[20.8,27.6]	33.9	[27.8,40.5]	37.2	[33.0,41.5]	11.4	[7.5,17.0]	
Student		1147	24.2	[21.5,27.3]	17.3	[13.2,22.3]	39.7	[35.4,44.3]	10.6	[6.9,15.9]	
Education level	4050										
Secondary not complete or less		2526	50.9	[47.4,54.5]	78.9	[73.5,83.4]	80.3	[76.7,83.6]	73.4	[64.5,80.7]	<0.0001
Secondary complete/higher		1524	49.1	[45.5,52.6]	21.1	[16.6,26.5]	19.7	[16.5,23.3]	26.6	[19.3,35.5]	
Past year sex partners	4013										
Never had sex, none, one		2965	74.3	[71.6,76.9]	75.5	[70.2,80.2]	71.1	[67.0,74.9]	68.8	[59.3,77.0]	0.2951
Two or more		1048	25.7	[23.2,28.4]	24.5	[19.8,29.8]	28.9	[25.1,33.0]	31.2	[23.0,40.7]	
Age sexual debut	4018										
< = 18		2077	56.5	[53.6,60.0]	56.6	[50.3,62.7]	51.8	[47.0,56.5]	47.1	[39.6,54.7]	<0.0001
> = 19		1076	28.3	[25.1,31.7]	31	[25.7,36.8]	22.4	[18.8,26.5]	39.4	[31.5,48.0]	
Never had sex		865	15.2	[13.3,17.4]	12.4	[9.3,16.2]	25.8	[21.8,30.3]	13.5	[8.2,21.4]	
**Table 1d: 2012 Survey: Men (15–49), socio-demographic and risk factors, by settlement type**											
Age	8510										
15–24		3486	32.8	[30.3,35.3]	26.7	[23.6,30.0]	43.5	[39.9,47.1]	24.8	[19.8,30.6]	<0.0001
25–49		5024	67.2	[64.7,69.7]	73.3	[70.0,76.4]	56.5	[52.9,60.1]	75.2	[69.4,80.2]	
Employment status	8091										
Employed		4103	55.8	[52.8,58.9]	50.3	[44.1,56.4]	26.2	[23.1,29.5]	75.6	[67.4,82.3]	<0.0001
Unemployed		2149	25.4	[23.0,28.0]	36.3	[31.4,41.5]	45.6	[42.4,49.0]	14.0	[8.8,21.6]	
Student		1839	18.7	[16.6,21.1]	13.4	[10.7,16.7]	28.1	[25.2,31.3]	10.4	[7.5,14.2]	
Education level	7544										
Secondary not complete or less		4446	46.5	[43.1,49.9]	70.4	[65.1,75.3]	74.6	[71.0,77.9]	62.2	[39.4,80.7]	<0.0001
Secondary complete/higher		3098	53.9	[50.6,57.1]	29.0	[24.2,34.3]	24.8	[21.5,28.4]	36.1	[19.9,56.2]	
Past year sex partners	8145										
Never had sex, none, one		7076	82.4	[79.8,84.7]	83.7	[79.1,87.4]	83.6	[80.8,86.0]	93.3	[90.4,95.4]	0.0024
Two or more		1069	17.6	[15.3,20.2]	16.3	[12.6,20.9]	16.4	[14.0,19.2]	6.7	[4.6,9.6]	
Age sexual debut	7715										
< = 18		3948	53.9	[51.0,56.8]	63.1	[58.4,67.6]	52.1	[48.6,55.6]	53.3	[48.4,58.3]	<0.0001
> = 19		2142	30.7	[28.0,33.5]	25.1	[20.7,30.1]	24.2	[21.1,27.6]	34.0	[29.8,38.5]	
Never had sex		1625	15.4	[13.6,17.4]	11.8	[8.9,15.4]	23.7	[21.3,26.2]	12.7	[9.2,17.3]	

Table 2 reports the 2012 survey for men, disaggregated by age groups. Among men 15–24, the largest proportion reporting being unemployed (34.6%) were in urban informal settlement and this was higher than urban formal (25.3%) and rural informal (27.4%) and significantly higher than rural formal (17.0%, p<0.001). Over a third (37.5%) of 15–24 year olds in urban formal settlements reported completed secondary education, compared to a quarter (24.4%) in urban informal, and rural formal (27.3%) and a fifth (17.3%) in rural informal, and this proportion was significantly larger (p<0.001). Age of sexual debut was significantly younger in urban settlements, with 56.5% in urban informal and 52.7% urban formal reporting this, compared to 45.3% in rural informal (p = 0.03).

**Table 2:** 2012 Survey for men socio-demographic and risk factors, by settlement type and age disaggregation.

**Table 2a: 2012 Survey: Men (15–24), socio-demographic and risk factors, by settlement type**											
			Urban Formal		Urban Informal		Rural Informal		Rural Formal		
	N	n	Col %	95% CI	Col %	95% CI	Col %	95% CI	Col %	95% CI	p-value
Employment status	3323										
Employed		704	21.1	[18.0, 24.5]	17.5	[12.3, 24.4]	8.4	[6.5, 10.7]	41.1	[14.6, 18.5]	<0.001
Unemployed		834	25.3	[22.0, 29.0]	34.6	[27.0, 43.0]	27.4	[24.0, 31.1]	17.0	[11.4, 24.5]	
Student		1785	53.6	[49.5, 57.7]	47.9	[38.2, 57.8]	64.2	[60.3, 60.0]	42.0	[30.4, 54.5]	
Education level	3149										
Secondary not complete or less		2252	62.5	[58.7, 66.2]	75.6	[67.9, 81.9]	82.7	[78.6, 86.2]	72.7	[58.7, 83.3]	<0.001
Secondary complete/higher		897	37.5	[33.8, 41.3]	24.4	[18.1, 32.1]	17.3	[13.8, 21.4]	27.3	[16.7, 41.4]	
Past year sex partners	3360										
Never had sex, none, one		2862	78.7	[74.5, 82.4]	77.7	[69.8, 84.0]	85.4	[82.2, 88.1]	83.0	[63.7, 93.1]	0.08
Two or more		498	21.3	[17.6, 25.5]	22.3	[16.0, 30.2]	14.6	[11.9, 17.8]	17.0	[6.9, 36.3]	
Age sexual debut	3291										
< = 18		1539	52.7	[48.8, 56.5]	56.5	[47.7, 64.8]	45.3	[41.4, 49.2]	51.4	[40.2, 62.5]	0.0323
> = 19		303	9.4	[7.4, 12.0]	7.4	[4.9, 11.1]	9.3	[7.2, 11.9]	11.7	[7.8, 17.1]	
Never had sex		1449	37.9	[34.3, 41.6]	36.1	[28.1, 45.0]	45.4	[41.7, 49.2]	36.9	[27.4, 47.5]	
**Table 2b: 2012 Survey: Men (25–49), socio-demographic and risk factors, by settlement type**											
Employment status	4768										
Employed		3399	72.3	[68.7, 75.7]	63.2	[55.9, 70.0]	40.6	[36.1, 45.2]	87.6	[77.6, 93.5]	<0.001
Unemployed		1315	25.2	[22.2, 28.6]	36.1	[29.2, 43.5]	58.7	[54.1, 63.2]	12.3	[6.4, 22.2]	
Student		54	2.4	[1.3, 4.4]	0.7	[0.3, 1.6]	0.7	[0.3, 1.6]	0.1	[0.03, 0.7]	
Education level	4395										
Secondary not complete or less		2194	38.4	[34.6, 42.4]	68.4	[61.5, 74.7]	67.8	[63.4, 71.9]	58.7	[34.1, 79.6]	<0.001
Secondary complete/higher		2201	61.6	[57.6, 65.4]	31.6	[25.3, 38.5]	32.2	[28.1, 36.6]	41.3	[20.4, 65.9]	
Past year sex partners	4785										
Never had sex, none, one		4214	84,2	[81.4, 86.7]	86.0	[81.4, 89.6]	82.1	[78.3, 85.4]	96.7	[93.9, 98.3]	0.0001
Two or more		571	15.8	[13.3, 18.6]	14.0	[10.4, 18.6]	17.9	[14.6, 21.7]	3.3	[1.7, 6.1]	
Age sexual debut	4424										
< = 18		2409	54.6	[50.8, 58.5]	65.9	[59.8, 71.4]	57.7	[52.4, 62.9]	54.0	[48.0, 60.0]	0.0042
> = 19		1839	42.3	[38.5, 46.2]	32.4	[26.6, 38.7]	36.4	[31.6, 41.5]	42.1	[36.0, 48.5]	
Never had sex		176	3.1	[2.0, 4.8]	1.8	[0.9, 3.5]	5.8	[4.1, 8.3]	3.8	[2.4, 6.2]	

For men aged 25–49 (Table 2B) the greatest proportion unemployed were in rural informal settlements (58.7%), and then urban informal settlements (36.1%), with significantly lower unemployment rates in urban formal (25.2%) and rural formal (12.3%, p<0.001). A significantly larger proportion of men in urban formal settlements (61.6%) had completed secondary education, compared to all other settlement types (31.6% urban informal; 32.2% rural informal; 41.3% rural formal, p<0.001). Finally, a greater proportion of men (25–49) in urban informal settlements (65.9%) reported sexual debut at 18 or less, compared to other settlement types (54.6% urban formal; 57.7% rural informal; 54.0% rural formal; p = 0.004).

HIV-prevalence by settlement type for men (15–49) showed consistent patterns (Table 3). HIV-prevalence in all surveys was highest in urban informal settlements, where it was almost twice that of urban formal settlements, and for 2005, 2008 and 2012, and these were significantly different. There was no difference between other settlement types, and urban formal settlements. Rural informal settlements showed an increasing HIV-prevalence over time (p = 0.001).

**Table 3:** Weighted HIV-prevalence for men over time by settlement type.

**Table 3a: Weighted HIV-prevalence for men (15–49) by settlement-type over time**					
	2002	2005	2008	2012	Trend test over time
	%(95%CI)	%(95%CI)	%(95%CI)	%(95%CI)	p-value
Urban formal	12.8 [9.9,16.3]	10.6 [8.3,13.6]	9.8 [7.4,12.7]	12.6 [10.3,15.5]	0.476
Urban informal	21.4 [14.7,30.0]	20 [15.3,25.8]	20.7 [16.0,26.4]	**22.6 [17.3,28.9]**	0.427
Rural informal	11.7 [8.4,16.1]	10.4 [7.8,13.7]	12.7 [9.2,17.3]	15.9 [13.6,18.5]	0.001
Rural formal	8 [4.6,13.6]	12.8 [7.3,21.4]	11.9 [7.1,19.2]	14 [8.3,22.5]	0.053
For year, by settlement type: p-value	0.0226	0.0318	0.0065	0.0122	
All estimates include adjustment for clustering and weighting of data					
**Table 3b: Weighted HIV-prevalence for men (15–24) by settlement-type for 2012**					
Urban formal	n/a	n/a	n/a	2.1 [1.3, 3.5]	
Urban informal	n/a	n/a	n/a	3.2 [1.6, 6.4]	
Rural informal	n/a	n/a	n/a	3.2 [1.9, 5.3]	
Rural formal	n/a	n/a	n/a	**8.2 [5.3, 12.5]**	
All estimates include adjustment for clustering and weighting of data					
**Table 3c: Weighted HIV-prevalence for men (25–49) by settlement-type for 2012**					
Urban formal	n/a	n/a	n/a	17.9 [14.2, 22.3]	
Urban informal	n/a	n/a	n/a	29.1 [22.2, 37.0]	
Rural informal	n/a	n/a	n/a	24.6 [20.6, 29.1]	
Rural formal	n/a	n/a	n/a	15.6 [8.8, 26.2]	

All estimates include adjustment for clustering and weighting of data

**Bolded** estimates indicate no overlap of 95% confidence intervals with urban formal settlements

In the age disaggregated tables (Table 3B and 3C) HIV prevalence was highest among 15–24 year olds residing in rural forma areas, followed by urban informal areas and rural informal areas. Among men 25–49 years old, the highest HIV-prevalence was among those in urban informal settlements, and lowest among those in urban formal settlements, though 95%CI did overlap.

Table 4 presents the unadjusted and adjusted relative risk ratios for all men by settlement type and survey year. In multivariable regressions (Table 4A), for all men (15–49) compared to urban formal settlements, urban informal settlements had a significantly higher relative risk for HIV-prevalence for 2005 (RR1.68, 1.11–2.50), 2008 (RR1.51, 1.04–2.18) and 2012 (RR1.77, 1.19–2.64). In 2002, men in rural formal settlements had a lower relative risk of HIV-prevalence than urban formal settlement (RR0.43, 0.22–0.84), while in 2012 men (15–49) in rural informal settings had a higher relative risk for HIV-prevalence (RR1.44, 1.05–1.97).

**Table 4:** Unadjusted and adjusted relative risk ratios for men for all surveys, by settlement type and in 2012 by age.

**Table 4a: Unadjusted and adjusted relative risk ratios for men (15–49) for all surveys, by settlement type and in 2012 by age.**								
	**2002 Men (15–49)**		**2005 Men (15–49)**		**2008 Men (15–49)**		**2012 Men (15–49)**	
	Unadjusted RR (95%CI)	Adjusted RR(95%CI)	Unadjusted RR (95%CI)	Adjusted RR(95%CI)	Unadjusted RR (95%CI)	Adjusted RR(95%CI)	Unadjusted RR (95%CI)	Adjusted RR(95%CI)
Urban formal	ref	ref	ref	ref	ref	ref	ref	ref
Urban informal	**1.67(1.08, 2.59)**	1.47(0.93, 2.31)	**1.88(1.31, 2.70)**	**1.68(1.11, 2.50)**	**2.12(1.47, 3.07)**	**1.51[1.04–2.18]**	**1.78(1.27, 2.52)**	**1.77[1.19, 2.64]**
Rural informal	0.92(0.61, 1.38)	1.19(0.76, 1.86)	0.98(0.67, 1.43)	1.06(0.67, 1.67)	1.30(0.86, 1.97)	1.29[0.83–2.02]	1.26(0.96, 1.65)	**1.44[1.05, 1.97]**
Rural formal	0.63(0.34, 1.14)	**0.43(0.22, 0.84)**	1.20(0.67, 2.18)	1.23(0.56, 2.70)	1.22(0.69, 2.15)	0.88[0.48–1.60]	1.12(0.66, 1.88)	1.13[0.62, 2.08]
	n = 2078, df = 768	n = 1933, df = 747	n = 3595, df = 815	n = 3064; df = 786	n = 3283, df = 886	n = 2961, df = 859	n = 6468, df = 880	n = 5109, df = 853
	p = 0.01	p<0.001	p = 0.002	p<0.001	p<0.001	p<0.001	p = 0.01	p<0.001
**Table 4b: Unadjusted and adjusted relative risk ratios for men in 2012 survey by age categories, and settlement type**							**2012 Men (15–24)**	
Urban formal	-	-	-	-	-	-	ref	ref
Urban informal	-	-	-	-	-	-	1.49(0.62, 3.56)	1.40[0.50–3.87]
Rural informal	-	-	-	-	-	-	1.48(0.72, 3.04)	**2.69[1.28–5.67]**
Rural formal	-	-	-	-	-	-	3.81(1.96, 7.41)	**3.59[1.49–8.64]**
	-	-	-	-	-	-	n = 2798, df = 757	n = 2347, df = 708
	-	-	-	-	-	-	p<0.001	p<0.001
							**2012 Men (25–49)**	
Urban formal	-	-	-	-	-	-		ref
Urban informal	-	-	-	-	-	-	1.62(1.15, 2.29)	**1.68[1.11–2.54]**
Rural informal	-	-	-	-	-	-	1.37(1.03, 1.83)	1.30[0.92–1.84]
Rural formal	-	-	-	-	-	-	0.87(0.48, 1.58)	1.00[0.51–1.99]
							n = 3670, df = 830	n = 2762, df = 780
							p = 0.02	p<0.001

All adjusted regressions are adjusted for: age, education, age first sexual debut, past year number of sexual partners.

All analyses include weighting and adjustment for study design

**Bolded** estimates indicate no overlap of 95% Confidence Intervals with reference category

In 2012, where it was possible to disaggregate men by age (Table 4), for men aged 15–24, compared to urban informal settlements, the relative risk of HIV-prevalence was higher in rural informal (RR2.69, 1.28–5.67) and rural formal (RR3.59, 1.49–8.64), compared to urban formal settlements, however confidence intervals were wide, highlighting lack of precision. And for men 25–49 in 2012, those in urban informal settlements had a higher relative risk of HIV-prevalence (RR1.68, 1.11–2.54) compared to urban formal settlements.

### Women

In total 3137, 7553, 6107 and 10454 women participated in the 2002, 2005, 2008, and 2012 surveys, respectively. In the 2002 survey, there were consistent patterns in distribution of socio-demographic and HIV-acquisition risk factors across both age groups (Table 5). Amongst all women (15–49; Table 5A) a significantly greater proportion of young women were in rural informal (46.6%) and rural formal (43.0%), compared to urban formal (31.0%) and urban informal (30.9%). For women in all age combinations (15–49; 15–24; 25–49) the highest proportion of unemployed were in urban informal settlements (Table 5A, 5B and 5C), and this was significantly higher than urban formal settlements (e.g. in 2002 for women 15–49 it was 68.9% compared to 47.4% for all women, respectively–Table 5A). The largest proportion who had not completed secondary school was in urban informal and rural informal for all age combinations, and the smallest proportion was always in urban formal settlements (Table 5A, 5B and 5C).

**Table 5:** Women (15–49) socio-demographic and risk factors, by settlement type for 2002.

**Table 5a: 2002 Survey: Women (15–49), socio-demographic and risk factors, by settlement type**											
			Urban Formal		Urban Informal		Rural Informal		Rural Formal		
	N	n	Col %	95% CI	Col %	95% CI	Col %	95% CI	Col %	95% CI	p-value
Age	3137										
15–24		1283	31.0	[28.3,33.8]	30.9	[25.6,36.7]	46.6	[42.4,51.0]	43.0	[32.1,54.7]	<0.0001
25–49		1854	69.0	[66.2,71.7]	69.1	[63.3,74.4]	53.4	[49.0,57.6]	57.0	[45.3,67.9]	
Employment status	2946										
Employed		846	37.1	[32.7,41.6]	19.1	[13.6,26.1]	10.6	[7.9,14.1]	29.4	[20.0,40.9]	<0.0001
Unemployed		1530	47.4	[42.7,52.0]	68.6	[59.3,76.5]	62.9	[57.2,68.2]	59.4	[45.7,71.8]	
Student		570	15.6	[13.4,18.0]	12.3	[8.5,17.6]	26.5	[22.1,31.4]	11.2	[5.2,22.4]	
Education level	3113										
Secondary not complete or less		2157	56.7	[51.5,61.6]	79.4	[71.3,85.7]	82.0	[77.6,85.7]	93.6	[83.6,97.7]	<0.0001
Secondary complete/higher		956	43.4	[38.4,48.5]	20.6	[14.3,28.7]	18.0	[14.3,22.4]	6.4	[2.3,16.4]	
Past year sex partners	3114										
Never had sex, none, one		3021	97.3	[95.9,98.2]	95.0	[90.6,97.4]	97.9	[95.5,99.1]	97.8	[94.3,99.2]	0.2749
Two or more		93	2.7	[1.8,4.1]	5.0	[2.6,9.4]	2.1	[0.9,4.5]	2.2	[0.8,5.7]	
Age sexual debut	3089										
< = 18		1374	42.3	[37.9,46.7]	61.9	[52.8,70.1]	50.1	[45.1,55.2]	58.5	[45.2,70.7]	<0.0001
> = 19		1134	43.5	[39.4,47.6]	28.2	[21.6,36.0]	27.3	[23.3,31.7]	26.8	[18.1,37.6]	
Never had sex		581	14.3	[12.1,16.8]	9.9	[6.2,15.5]	22.6	[18.8,26.9]	14.7	[6.6,29.6]	
**Table 5b: 2002 Survey: Women (15–24), socio-demographic and risk factors, by settlement type**											
Employment status	1176										
Employed		143	13.7	[8.6,21.2]	6.5	[2.7,14.6]	2.1	[0.9,4.6]	15.4	[8.1,27.3]	<0.0001
Unemployed		469	35.8	[29.2,43.0]	54.7	[43.4,65.4]	40.2	[33.2,47.8]	56.6	[38.2,73.3]	
Student		564	50.5	[43.6,57.4]	38.9	[28.3,50.6]	57.7	[50.1,64.9]	28.0	[13.3,49.7]	
Education level	1268										
Secondary not complete or less		176	55.9	[49.4,62.1]	74.3	[64.0,82.5]	81.2	[74.8,86.2]	97.0	[91.6,99.0]	<0.0001
Secondary complete/higher		400	44.1	[37.9,50.6]	25.7	[17.6,36.0]	18.8	[13.8,25.2]	3.0	[1.0,8.4]	
Past year sex partners	1271										
Never had sex, none, one		1215	94.7	[91.1,96.9]	89.0	[76.5,95.2]	98.3	[96.5,99.2]	97.9	[92.3,99.5]	0.002
Two or more		56	5.3	[3.1,8.9]	11.0	[4.8,23.5]	1.7	[0.8,3.5]	2.1	[0.5,7.7]	
Age sexual debut	1260										
< = 18		541	42.2	[35.7,48.9]	58.7	[44.6,71.5]	43.7	[36.4,51.2]	58.0	[37.1,76.3]	0.225
> = 19		180	15.8	[11.7,21.0]	12.5	[5.3,26.4]	10.9	[7.1,16.4]	8.3	[3.9,16.8]	
Never had sex		539	42.0	[35.7,48.7]	28.8	[17.8,43.0]	45.4	[38.6,52.3]	33.7	[16.1,57.5]	
**Table 5c: 2002 Survey: Women (25–49), socio-demographic and risk factors, by settlement type**											
Employment status	1770										
Employed		703	47.3	[41.9,52.8]	24.9	[16.4,35.9]	17.9	[13.0,24.0]	38.7	[23.4,56.6]	<0.0001
Unemployed		1061	52.5	[47.0,57.9]	74.9	[63.9,83.5]	82.1	[76.0,87.0]	61.3	[43.4,76.6]	
Student		6	0.2	[0.0,0.8]	0.2	[0.0,1.3]	0		0		
Education level	1845										
Secondary not complete or less		1289	57	[50.8,63.0]	81.7	[71.3,88.9]	82.7	[76.0,87.8]	91	[72.4,97.5]	<0.0001
Secondary complete/higher		556	43	[37.0,49.2]	18.3	[11.1,28.7]	17.3	[12.2,24.0]	9	[2.5,27.6]	
Past year sex partners	1843										
Never had sex, none, one		1806	98.4	[96.9,99.2]	97.7	[94.3,99.1]	97.6	[92.4,99.3]	97.7	[91.3,99.4]	0.8176
Two or more		37	1.6	[0.8,3.1]	2.3	[0.9,5.7]	2.4	[0.7,7.6]	2.3	[0.6,8.7]	
Age sexual debut	1829										
< = 18		833	42.3	[37.3,47.5]	63.2	[53.0,72.4]	55.8	[49.3,62.1]	59	[42.6,73.6]	0.0005
> = 19		954	55.9	[50.8,60.9]	35.2	[26.1,45.4]	41.7	[35.5,48.2]	40.6	[26.1,57.0]	
Never had sex		42	1.8	[0.9,3.7]	1.6	[0.7,3.7]	2.5	[1.1,5.9]	0.4	[0.1,3.0]	

Consistently, over the three age categorization the largest proportion reporting sexual debut ≤18 years, was in urban informal settlements, and smallest in urban formal settlements. For the combined group (15–49) and older women (25–49) this was significantly different. For women aged 15–24 (Table 5C), a greater proportion in urban informal settlements reported ≥2 past year sexual partners (11.0%) and this was significantly different to other settlement types, where it was <5.0%.

The other three surveys showed very similar patterns in the distribution of socio-demographics and risk factors as the 2002 survey (Tables 6, 7 and 8). For example the 2012 survey showed very similar patterns for all women (15–49), adolescent women (15–24) and older women (25–49) (Table 8A, 8B and 8C respectively), as the 2002 survey. This included the largest proportion of young (15–24) women being in rural informal settlements (39.3%). The highest proportion of unemployed women was in urban informal and rural informal for all age combinations; for instance amongst 15–24 year olds, half (50.8%) in urban informal reported being unemployed, compared to 29.0% in urban formal settlements. Amongst all women, there were no differences with education levels, but among 15–24 nearly three-quarters in urban informal (73.7%), and rural informal (74.0%) had not completed secondary school, compared to half (53.5%) in urban formal.

**Table 6:** Women (15–49) socio-demographic and risk factors, by settlement type for 2005.

**Table 6a: 2005 Survey: Women (15–49), socio-demographic and risk factors, by settlement type**											
			Urban Formal		Urban Informal		Rural Informal		Rural Formal		
	N	n	Col %	95% CI	Col %	95% CI	Col %	95% CI	Col %	95% CI	p-value
Age	7553										
15–24		3171	31.2	[28.9,33.6]	33.7	[30.1,37.5]	38.5	[35.3,41.7]	35.7	[31.9,39.6]	<0.0001
25–49		4382	68.8	[66.4,71.1]	66.3	[62.5,69.9]	61.5	[58.3,64.7]	64.3	[60.4,68.1]	
Employment status	7356										
Employed		2099	36.7	[33.8,39.7]	23	[18.7,28.0]	12.2	[10.3,14.5]	36.3	[29.3,43.9]	<0.0001
Unemployed		3693	45.7	[42.4,48.9]	63.3	[57.0,69.1]	66.2	[63.1,69.2]	55.1	[47.8,62.2]	
Student		1564	17.6	[15.5,20.0]	13.7	[10.8,17.2]	21.6	[19.2,24.1]	8.6	[6.3,11.7]	
Education level	7475										
Secondary not complete or less		4800	52.7	[48.9,56.5]	78.1	[73.6,82.1]	74.2	[70.9,77.3]	87.2	[82.7,90.7]	<0.0001
Secondary complete/higher		2675	47.3	[43.5,51.1]	21.9	[17.9,26.4]	25.8	[22.7,29.1]	12.8	[9.3,17.3]	
Past year sex partners	7223										
Never had sex, none, one		7090	97.5	[96.6,98.2]	97.4	[95.4,98.6]	98.7	[97.9,99.1]	98.8	[97.4,99.4]	0.0424
Two or more		133	2.5	[1.8,3.4]	2.6	[1.4,4.6]	1.3	[0.9,2.1]	1.2	[0.6,2.6]	
Age sexual debut	6762										
< = 18		3143	43.6	[40.5,46.7]	62.1	[56.7,67.2]	54.2	[50.0,58.3]	63.4	[57.4,69.0]	<0.0001
> = 19		2275	40.8	[38.0,43.6]	27	[23.2,31.1]	29.1	[25.5,32.9]	27.5	[22.6,33.0]	
Never had sex		1344	15.7	[13.8,17.8]	11	[7.7,15.3]	16.8	[14.7,19.0]	9.1	[6.7,12.4]	
	**Table 6b: 2005 Survey: Women (15–24), socio-demographic and risk factors, by settlement type**										
Employment status	3096										
Employed		384	14.7	[12.0,17.9]	8.7	[5.7,13.1]	1.7	[1.0,2.8]	21.3	[15.1,29.1]	<0.0001
Unemployed		1180	31.7	[27.5,36.3]	51.9	[44.0,59.7]	43.7	[39.4,48.0]	55.1	[48.0,62.0]	
Student		1532	53.5	[49.2,57.8]	39.4	[32.4,46.8]	54.6	[50.3,58.9]	23.6	[17.7,30.9]	
Education level	3135										
Secondary not complete or less		2037	58.2	[53.7,62.6]	77.8	[70.5,83.7]	71.8	[67.5,75.7]	86	[79.6,90.6]	<0.0001
Secondary complete/higher		1098	41.8	[37.4,46.3]	22.2	[16.3,29.5]	28.2	[24.3,32.5]	14	[9.4,20.4]	
Past year sex partners	3027										
Never had sex, none, one		2948	96.1	[94.5,97.3]	95.1	[89.6,97.7]	98.1	[96.8,98.9]	97.8	[94.3,99.2]	0.0542
Two or more		79	3.9	[2.7,5.5]	4.9	[2.3,10.4]	1.9	[1.1,3.2]	2.2	[0.8,5.8]	
Age sexual debut	2978										
< = 18		1284	39.4	[35.0,44.1]	60.7	[50.9,69.7]	47.5	[42.1,53.0]	60.3	[51.4,68.6]	0.0003
> = 19		434	19	[15.3,23.2]	12.4	[8.6,17.5]	14.2	[10.5,18.9]	15.8	[9.7,24.7]	
Never had sex		1260	41.6	[37.5,45.8]	27	[19.4,36.2]	38.3	[34.6,42.2]	23.9	[18.1,31.0]	
	**Table 6c: 2005 Survey: Women (25–49), socio-demographic and risk factors, by settlement type**										
Employment status	4260										
Employed		1715	46.7	[42.9,50.5]	30.4	[24.3,37.3]	19	[15.8,22.5]	44.9	[35.5,54.6]	<0.0001
Unemployed		2513	52	[48.1,55.8]	69.1	[62.2,75.2]	80.5	[76.9,83.7]	55.1	[45.4,64.5]	
Student		32	1.3	[0.7,2.5]	0.5	[0.2,1.4]	0.5	[0.3,1.1]	0		
Education level	4340										
Secondary not complete or less		2763	50.2	[45.8,54.6]	78.3	[73.6,82.3]	75.8	[71.2,79.9]	87.9	[82.0,92.1]	<0.0001
Secondary complete/higher		1577	49.8	[45.4,54.2]	21.7	[17.7,26.4]	24.2	[20.1,28.8]	12.1	[7.9,18.1]	
Past year sex partners	4196										
Never had sex, none, one		4142	98.2	[96.9,98.9]	98.6	[96.9,99.4]	99	[98.0,99.5]	99.3	[97.999.8]	0.2564
Two or more		54	1.8	[1.1,3.1]	1.4	[0.6,3.1]	1	[0.5,2.0]	0.7	[0.2,2.1]	
Age sexual debut	3784										
< = 18		1859	45.5	[42.1,49.0]	62.8	[57.1,68.2]	59	[53.9,64.0]	65.2	[58.0,71.8]	<0.0001
> = 19		1841	51.1	[47.6,54.6]	34.9	[29.6,40.6]	40	[35.1,45.0]	34.6	[28.1,41.8]	
Never had sex		84	3.4	[2.3,5.0]	2.3	[1.0,5.5]	1	[0.4,2.4]	0.2	[0.03,1.3]	

**Table 7:** Women (15–49) socio-demographic and risk factors, by settlement type for 2008.

	**Table 7a: 2008 Survey: Women (15–49), socio-demographic and risk factors, by settlement type**										
	N	n	Urban Formal		Urban Informal		Rural Informal		Rural Formal		
			Col %	95% CI	Col %	95% CI	Col %	95% CI	Col %	95% CI	
Age	6107										
15–24		2465	30.5	[28.2,32.9]	33.1	[29.9,36.5]	41.6	[39.1,44.2]	26.5	[22.7,30.8]	<0.0001
25–49		3642	69.5	[67.1,71.8]	66.9	[63.5,70.1]	58.4	[55.8,61.0]	73.5	[69.2,77.3]	
Employment status	5682										
Employed		1933	44.7	[41.4,48.0]	21.3	[17.1,26.0]	12.7	[10.0,16.1]	37.7	[30.3,45.8]	<0.0001
Unemployed		2630	40.3	[37.3,43.5]	63.9	[59.2,68.4]	63.8	[60.1,67.4]	53.5	[46.0,60.9]	
Student		1119	15	[13.5,16.7]	14.8	[11.9,18.3]	23.5	[20.9,26.3]	8.7	[5.7,13.2]	
Education level	5770										
Secondary not complete or less		3542	47.4	[44.1,50.7]	73.2	[67.9,78.0]	75.1	[71.2,75.1]	68.5	[61.1,75.1]	<0.0001
Secondary complete/higher		2228	52.6	[49.3,55.9]	26.8	[22.1,32.1]	24.9	[21.3,28.8]	31.5	[24.9,38.9]	
Past year sex partners	5730										
Never had sex, none, one		4524	80	[77.6,82.3]	79.6	[74.8,83.6]	76.4	[73.1,79.4]	81.7	[75.6,86.6]	0.1781
Two or more		1206	20	[17.7,22.4]	20.4	[16.4,25.2]	23.6	[20.6,26.9]	18.3	[13.4,24.4]	
Age sexual debut	5731										
< = 18		2793	46.4	[43.5,49.3]	60.6	[56.2,64.8]	57.3	[53.6,60.8]	53.8	[46.7,60.8]	<0.0001
> = 19		2101	41.7	[38.9,44.6]	30.8	[27.3,34.5]	31.4	[28.1,34.8]	40.2	[33.3,47.5]	
Never had sex		837	11.9	[10.4,13.7]	8.7	[6.6,11.4]	11.4	[9.5,13.6]	6	[4.0,8.8]	
	**Table 7b: 2008 Survey: Women (15–24), socio-demographic and risk factors, by settlement type**										
Employment status	2236										
Employed		335	15.7	[12.6,19.4]	5.1	[3.0,8.6]	3.6	[2.0,6.7]	19.1	[12.9,27.3]	<0.0001
Unemployed		802	34.9	[30.2,40.0]	50	[42.3,57.7]	39.2	[34.2,44.4]	49.3	[38.2,60.5]	
Student		1099	49.4	[44.4,54.5]	44.9	[37.3,52.8]	57.2	[51.9,62.3]	31.6	[22.7,42.2]	
Education level	2254										
Secondary not complete or less		1467	58.6	[53.6,63.5]	71.5	[64.2,77.9]	75.7	[71.1,79.8]	71.7	[61.3,70.1]	<0.0001
Secondary complete/higher		787	41.4	[36.5,46.4]	28.5	[22.2,35.8]	24.3	[20.2,28.9]	28.3	[19.8,38.7]	
Past year sex partners	2255										
Never had sex, none, one		1869	85.2	[81.7,88.0]	82.1	[76.3,86.8]	76.8	[72.0,81.0]	80.3	[72.2,86.5]	0.0045
Two or more		386	14.9	[12.0,18.3]	17.9	[13.3,23.7]	23.2	[19.0,28.0]	19.7	[13.5,27.8]	
Age sexual debut	2254										
< = 18		1053	42.8	[38.4,47.3]	56.1	[49.0,63.0]	54.5	[49.4,59.5]	54.1	[44.2,63.7]	0.0005
> = 19		364	17.3	[13.7,21.6]	17.6	[13.1,23.2]	16.8	[13.4,21.0]	21.5	[14.3,31.1]	
Never had sex		837	39.9	[35.2,44.8]	26.3	[20.6,32.9]	28.7	[24.2,33.7]	24.3	[16.7,34.0]	
	**Table 7c: 2008 Survey: Women (25–49), socio-demographic and risk factors, by settlement type**										
Employment status	3446										
Employed		1598	57	[53.0,60.9]	29.2	[23.5,35.6]	18.7	[14.8,23.3]	43.9	[35.0,53.2]	<0.0001
Unemployed		1828	42.6	[38.7,46.6]	70.8	[64.4,76.5]	80.1	[75.6,84.0]	54.9	[45.7,63.8]	
Student		20	0.4	[0.2,0.9]	0		1.2	[0.5,2.9]	1.2	[0.2,7.7]	
Education level	3516										
Secondary not complete or less		2075	42.7	[38.7,46.8]	74	[68.1,79.2]	74.8	[69.7,79.2]	67.5	[58.0,75.7]	<0.0001
Secondary complete/higher		1441	57.3	[53.3,61.3]	26	[20.8,31.9]	25.2	[20.8,31.9]	32.5	[24.3,42.0]	
Past year sex partners	3457										
Never had sex, none, one		2655	77.8	[74.8,80.5]	78.3	[71.8,83.7]	76.1	[72.0,79.7]	82.2	[74.6,87.9]	0.5178
Two or more		820	22.2	[19.5,25.2]	21.7	[16.3,28.2]	23.9	[20.3,28.0]	17.8	[12.1,25.4]	
Age sexual debut	3477										
< = 18		1740	47.9	[44.3,51.5]	62.7	[57.7,67.5]	59.1	[54.1,63.9]	53.7	[44.8,62.5]	<0.0001
> = 19		1737	52.1	[48.5,55.7]	37.3	[32.5,42.3]	40.9	[36.1,45.9]	46.3	[37.5,55.3]	
Never had sex											

**Table 8:** Women (15–49) socio-demographic and risk factors, by settlement type for 2012.

**Table 8a: 2012 Survey: Women (15–49), socio-demographic and risk factors, by settlement type**											
			Urban formal		Urban informal		Rural informal		Rural formal		
** **	N	n	Col %	95% CI	Col %	95% CI	Col %	95% CI	Col %	95% CI	
Age	10454										
15–24		3733	32.5	[30.5,34.4]	29.9	[26.8,33.1]	39.3	[36.8,41.8]	28.5	[22.5,35.3]	<0.0001
25–49		6721	67.5	[65.6,69.5]	70.1	[66.9,73.2]	60.7	[58.2,63.2]	71.5	[64.7,77.5]	
Employment status	9988										
Employed		3539	42.9	[39.8,46.0]	31.4	[28.3,34.6]	17.8	[15.1,20.8]	43.1	[37.3,49.0]	<0.0001
Unemployed		4540	38.8	[35.5,42.1]	55.7	[52.7,58.7]	57.9	[54.9,60.8]	43.2	[37.5,49.1]	
Student		1909	18.4	[16.5,20.4]	12.9	[10.4,16.0]	24.3	[22.4,26.4]	13.7	[8.1,22.4]	
Education level	9223										
Secondary not complete or less		5173	45.4	[41.6,49.2]	69.8	[64.7,74.4]	68.9	[65.5,72.1]	67.2	[59.4,74.1]	<0.0001
Secondary complete/higher		4050	54.6	[50.8,58.4]	30.2	[25.6,35.3]	31.1	[27.9,34.5]	32.8	[25.9,40.6]	
Past year sex partners	10054										
Never had sex, none, one		9737	96.0	[95.0,96.8]	95.6	[93.6,97.0]	97.0	[96.1,97.8]	98.2	[96.6,99.1]	0.0607
Two or more		315	4.0	[3.2,5.0]	4.4	[3.0,6.4]	3.0	[2.2,3.9]	1.8	[0.9,3.4]	
Age sexual debut	9692										
< = 18		4531	47.6	[44.6,50.6]	64.0	[59.4,68.4]	51.6	[48.9,54.3]	51.9	[45.9,57.9]	<0.0001
> = 19		3540	38.2	[35.5,40.9]	26.6	[22.6,31.0]	31.5	[28.7,34.4]	31.8	[27.6,36.3]	
Never had sex		1621	14.3	[12.8, 16.0]	9.3	[7.3, 11.9]	16.9	[15.0, 18.9]	16.3	[11.3, 23.0]	
**Table 8b: 2012 Survey: Women (15–24), socio-demographic and risk factors, by settlement type**											
Employment status	3572										
Employed		486	18.1	[14.4,22.5]	9.2	[5.9,14.1]	5.3	[3.7,7.6]	15.6	[9.8,23.9]	<0.0001
Unemployed		1261	29	[25.3,33.0]	50.8	[44.1,57.4]	35.3	[32.0,38.7]	36.6	[26.1,48.6]	
Student		1825	52.9	[48.3,57.6]	40	[33.4,46.9]	59.4	[55.5,63.2]	47.8	[33.5,62.6]	
Education level	3353										
Secondary not complete or less		2156	53.5	[49.1,57.9]	73.7	[66.5,79.8]	74	[69.4,78.1]	63.2	[47.1,76.7]	<0.0001
Secondary complete/higher		1197	46.5	[42.1,50.8]	27.2	[21.0,34.4]	25.2	[21.0,30.0]	38.8	[23.7,56.3]	
Past year sex partners	3596										
Never had sex, none, one		3449	95.2	[93.5,96.5]	91.1	[85.2,94.7]	96.7	[94.9,97.9]	98.8	[96.1,99.6]	0.0078
Two or more		147	4.8	[3.5,6.5]	9	[5.3,14.8]	3.3	[2.1,5.1]	1.2	[0.4,3.9]	
Age sexual debut	3562										
< = 18		1569	45.9	[41.6,50.2]	63	[56.0,69.4]	45	[41.0,49.1]	46.9	[34.4,59.7]	0.008
> = 19		525	16.7	[14.1,19.7]	9.1	[6.3,13.0]	15.7	[12.8,19.0]	10.3	[6.9,15.1]	
Never had sex		1468	37.4	[33.5,41.5]	28	[22.5,34.2]	39.3	[35.9,42.9]	42.8	[30.5,56.0]	
**Table 8c: 2012 Survey: Women (25–49), socio-demographic and risk factors, by settlement type**											
Employment status	6416										
Employed		3053	54.8	[50.9,58.6]	40.9	[36.8,45.0]	26	[22.0,30.5]	54.1	[48.6,59.5]	<0.0001
Unemployed		3279	43.5	[39.6,47.5]	57.8	[53.3,62.2]	72.7	[68.3,76.8]	45.9	[40.5,51.4]	
Student		84	1.7	[1.2,2.5]	1.3	[0.5,3.3]	1.3	[0.6,2.5]	0		
Education level	5870										
Secondary not complete or less		3017	41.3	[36.9,45.8]	68	[62.7,72.9]	65.3	[61.0,69.3]	68.9	[60.0,76.6]	<0.0001
Secondary complete/higher		2853	58.7	[54.2,63.1]	32	[27.1,37.3]	34.7	[30.7,39.0]	31.1	[23.4,40.0]	
Past year sex partners	6456										
Never had sex, none, one		6288	96.4	[95.0,97.4]	97.5	[95.7,98.6]	97.2	[95.9,98.1]	98	[95.7,99.1]	0.3502
Two or more		168	3.6	[2.6,5.0]	2.5	[1.4,4.3]	2.8	[1.9,4.1]	2	[0.9,4.3]	
Age sexual debut	6130										
< = 18		2962	48.5	[45.0,52.0]	64.5	[58.8,69.9]	56.2	[52.3,60.0]	54.1	[46.0,61.9]	0.0015
> = 19		3015	49.4	[45.9,52.9]	34.5	[29.1,40.4]	42.4	[38.6,46.1]	40.8	[35.8,46.1]	
Never had sex		153	2.1	[1.6, 2.9]	1	[0.4, 2.2]	1.5	[0.7, 3.1]	5.1	[1.3, 18.6]	

For HIV-risk factors in the 2012 survey, amongst those 15–24 (Table 8B), the greatest proportion reporting two or more past year sexual partners were those in urban informal settlements (9.0%) and this was significantly different to other settlement types (4.8% urban formal; 3.3% rural informal; 1.2% rural formal; p = 0.008). For all age combinations, the greatest proportion reporting sexual debut of ≤18 years was in urban informal settlements, and lowest proportion in urban formal settlements, and these were all significantly different.

HIV-prevalence by settlement types, over surveys for all age categorisations for women (Table 9) showed consistent patterns. The highest HIV-prevalence was among urban informal settlements. For women 15–49 (Table 9A), compared to urban formal settlements these differences were significant at all-time points, and in 2008 and 2012, compared to urban formal settlements, rural informal settlements were also significantly higher. Similarly for women 15–24 (Table 9B), compared to urban formal settlements, urban informal settlements had a significantly higher HIV-prevalence for 2005, 2008 and 2012. And for women 25–49 (Table 9C), urban informal settlements had a higher proportion of HIV-positive women, in all years, and in 2008 and 2012, in rural informal areas also had a higher proportion of HIV-positive women, compared to urban formal areas.

**Table 9:** Weighted HIV-prevalence for women by settlement type over time.

**Table 9a Weighted HIV-prevalence for women (15–49) by settlement-type over time **					
	2002	2005	2008	2012	Time-trend
	%(95%CI)	%(95%CI)	%(95%CI)	%(95%CI)	p-value
Urban formal	18.1 [14.8,22.0]	17.2 [14.5,20.3]	16.8 [14.2,19.7]	16.9 [14.2,19.9]	0.308
Urban informal	**33.8 [24.4,44.8]**	**30.7 [26.8,35.0]**	**34.6 [30.4,39.0]**	**38.1 [34.0,42.3]**	0.006
Rural informal	12.9 [9.5,17.3]	21.8 [18.4,25.7]	**23.9 [20.6,27.6]**	**29.4 [26.5,32.5]**	<0.0001
Rural formal	15.2 [7.6,28.3]	15.5 [10.9,21.5]	21.4 [16.4,27.5]	20.0 [15.0,26.2]	0.027
For year, by settlement type: p-value	0.0003	0.0001	<0.0001	<0.0001	
**Table 9b: Weighted HIV-prevalence for women (15–24), by settlement-type over time**					
Urban formal	13.7 [9.7,19.2]	12.3 [9.4,16.1]	11.3 [8.5,14.9]	9.4 [7.3,12.0]	0.001
Urban informal	28.3 [17.9,41.7]	**30.7 [24.9,37.1]**	**21.0 [16.1,26.8]**	**18.9 [13.1,26.5]**	0.001
Rural informal	7.5 [4.7,11.8]	18.4 [14.2,23.4]	14.0 [10.5,18.4]	12.6 [9.8,16.0]	0.928
Rural formal	10.1 [4.2,22.5]	16.7 [10.6,25.2]	19.2 [12.8,27.9]	10.7 [7.5,15.1]	0.419
For year, by settlement type: p-value	0.0006	0.0002	0.018	0.0174	
**Table 9c: Weighted HIV-prevalence for women (25–49), by settlement-type over time**					
Urban formal	20.1 [15.9,25.2]	19.4 [16.1,23.2]	19.2 [15.9,23.0]	20.5 [16.9,24.7]	0.565
Urban informal	**36.2 [25.5,48.6]**	**30.8 [25.8,36.2]**	**41.8 [35.8,48.1]**	**45.8 [41.1,50.5]**	<0.0001
Rural informal	17.8 [12.5,24.7]	23.9 [19.8,28.6]	**31.7 [26.9,36.9]**	**40.2 [36.3,44.2]**	<0.0001
Rural formal	18.5 [8.2,36.5]	14.9 [9.6,22.4]	22.3 [15.8,30.5]	23.4 [16.9,31.4]	0.013
For year, by settlement type: p-value	0.0296	0.0049	<0.0001	<0.0001	

All estimates include adjustment for clustering and weighting of data

**Bolded** estimates indicate no overlap of 95% confidence intervals with urban informal settlements

In the combined age group for women (15–49; Table 9A) urban informal, rural informal and rural formal, all saw significantly increasing HIV-prevalence trends. In younger (15–24; Table 9B) women, there were decreasing HIV-prevalence trends for urban formal and urban informal. And for older (25–49; Table 9C) women, increasing HIV-prevalence trends for urban informal, rural informal and rural formal settlement types.

In adjusted analyses for women (Table 10), for all survey years, and in all age categorisations (15–49; 15–24; 25–49), compared to urban formal settlements, women in urban informal settlements had significantly higher relative risks of living with HIV. In other settlement types, compared to urban formal settlements, there was no consistent patterning, but in 2012, women in rural informal settlements had a higher relative risk for HIV-prevalence, for all age combinations (15–49; 15–24; 25–49).

**Table 10:** Unadjusted and adjusted relative risk ratios for women by settlement type, and survey.

**Table 10a: Unadjusted and adjusted relative risk ratios for women (15–49), by settlement type, and survey**								
	**2002 Women (15–49)**		**2005 Women (15–49)**		**2008 Women (15–49)**		**2012 Women (15–49)**	
	Unadjusted RR (95%CI)	Adjusted RR(95%CI)	Unadjusted RR (95%CI)	Adjusted RR(95%CI)	Unadjusted RR (95%CI)	Adjusted RR(95%CI)	Unadjusted RR (95%CI)	Adjusted RR(95%CI)
Urban formal	ref	ref	ref	ref	ref	ref	ref	ref
Urban informal	**1.87(1.30, 2.68)**	**1.75(1.23, 2.48)**	**1.79(1.44, 2.22)**	**1.64(1.25, 2.14)**	**2.06(1.68, 2.53)**	**1.64[1.32–2.03]**	**2.26(1.83, 2.78)**	**1.89[1.50–2.40]**
Rural informal	0.71(0.50, 1.02)	0.74(0.50, 1.10)	**1.27(1.00, 1.61)**	1.22(0.93, 1.59)	**1.43(1.14, 1.78)**	1.13[0.89–1.43]	**1.72(1.40, 2.11)**	**1.60[1.27, 1.99]**
Rural formal	0.84(0.42, 1.69)	0.98(0.50, 1.91)	0.90(0.62, 1.32)	0.81(0.50, 1.31)	1.28(0.94, 1.74)	1.04[0.77–1.42]	1.14(0.82, 1.60)	0.95[0.65–1.38]
	n = 2717; df = 818	n = 2495, df = 808	n = 5650, df = 837	n = 4910, df = 824	n = 4823, df = 923	n = 4363, df = 905	n = 8253, df = 909	n = 6650, df = 883
	p<0.001	p<0.001	p<0.001	p<0.001	p<0.001	p<0.001	p<0.001	p<0.001
**Table 10b: Unadjusted and adjusted relative risk ratios for women (15–24), by settlement type, and survey**								
	**2002 Women (15–24)**		**2005 Women (15–24)**		**2008 Women (15–24)**		**2012 Women (15–24)**	
Urban formal	ref	ref	ref	ref	ref	ref	ref	ref
Urban informal	**2.06(1.19, 3.56)**	**1.86(1.10, 3.14)**	**2.49(1.78, 3.49)**	**2.07(1.43, 2.99)**	**1.85(1.27, 2.70)**	**1.50[1.02–2.22]**	**2.07(1.34, 3.19)**	**1.79[1.17–2.73]**
Rural informal	**0.55(0.31, 0.97)**	0.63(0.33, 1.21)	**1.49(1.02, 2.16)**	1.41(0.95 2.08)	1.23(0.83, 1.84)	0.92[0.61–1.41]	1.38(0.98, 1.95)	**1.45[1.01–2.08]**
Rural formal	0.74(0.29, 1.85)	0.88(0.34 2.28)	1.35(0.81, 2.26)	1.13(0.65, 1.95)	1.70(1.04, 2.75)	1.07[0.68–1.68]	1.12(0.71, 1.74)	0.93[0.54–1.60]
	n = 1123, df = 599	n = 1004, df = 563	n = 2335, df = 717	n = 2134, df = 695	n = 1986, df = 745	n = 1754, df = 674	n = 3092, df = 782	n = 2586, df = 741
	p<0.001	p<0.001	p<0.001	p<0.001	p = 0.01	p<0.001	p<0.001	p<0.001
**Table 10c: Unadjusted and adjusted relative risk ratios for women (25–49), by settlement type, and survey**								
	**2002 Women (25–49)**		**2005 Women (25–49)**		**2008 Women (25–49)**		**2012 Women (25–49)**	
Urban formal	ref	ref	ref	ref	ref	ref	ref	ref
Urban informal	**1.80(1.21, 2.68)**	**1.68(1.11, 2.53)**	**1.58(1.24, 2.03)**	**1.48(1.09, 2.02)**	**2.18(1.72, 2.77)**	**1.66[1.31–2.12]**	**2.23(1.76, 2.82)**	**1.91[1.47–2.48]**
Rural informal	0.88(0.58, 1.34)	0.82(0.51 1.31)	1.23(0.95, 1.60)	1.15(0.85, 1.56)	**1.65(1.30, 2.12)**	1.20[0.92–1.57]	**1.90(1.52, 2.39)**	**1.67[1.31–2.14]**
Rural formal	0.92(0.41, 2.03)	1.12(0.52, 2.40)	0.77(0.48, 1.22)	0.68(0.36, 1.28)	1.16(0.80, 1.70)	1.07[0.73–1.56]	1.10(0.76, 1.60)	1.00[0.67–1.49]
	n = 1594, df = 729	n = 1491, df = 716	n = 3315, df = 813	n = 2776, df = 796	n = 2837, df = 882	n = 2609, df = 855	n = 5161, df = 886	n = 4064, df = 850
	p = 0.01	p<0.001	p<0.001	p<0.001	p<0.001	p<0.001	p<0.001	p<0.001

All adjusted regressions are adjusted for: age, education, age first sexual debut, past year number of sexual partners.

All analyses include weighting and adjustment for study design

**Bolded** estimates indicate no overlap of 95% Confidence Intervals with reference category

## Discussion

Our analysis of four representative household surveys in South Africa from 2002 to 2012, show consistent patterning around the distribution of HIV, by settlement types, with urban informal settlements showing the highest prevalence. This finding was consistent in multivariate models accounting for potential socio-demographic cofounders, and in multivariate models for all women and men aged 15–49, and for women across different age stratifications (15–24; 25–49). For men, stratification by age was only possible for the most recent survey (2012), and in adjusted models urban informal settlements had the highest relative risk of HIV-prevalence for older men (25–49), but for younger men (15–24) adjusted relative risk HIV-prevalence was highest among rural formal settlements.

The markedly high HIV-prevalence, and consistent association between urban informal settlements and HIV-prevalence, even in adjusted models may have a number of underlying explanations. HIV-risk behaviour showed clear patterning by settlement type, with early sexual debut more common amongst both women and men residing in urban informal settlements across all surveys (and age categorisations). Similarly, young women (15–24) in all survey waves more often reported two or more past year sexual partners in urban informal settlements compared to other settlement types. Early sexual debut and multiple sexual partners, both of which are often driven by poverty, inequitable gender norms and violence, are key risks for HIV-acquisition [21–23]. Interventions targeting these factors may be important strategies.

Structural factors may also shape the disproportionate burden of HIV in urban informal settlements. Across surveys, women and men in urban informal settlements had lower educational attainment than those in formal settlements. Low education, since the early 2000s, has been recognised as a risk factor for HIV [24]. Similarly, urban informal settlements had high unemployment rates, and unemployment, via increased poverty, may be a risk factor for HIV-acquisition [1]. Structural interventions focused on strengthening educational attainment, and increasing employment/reducing poverty, are potentially important interventions.

Even after adjustment for potential co-founders, informal settlements consistently had higher HIV-prevalence than other settlement types, suggesting there remained unmeasured factors. Housing in informal settlements can be unstable, and studies from North America have highlighted how housing instability is an independent risk factor for HIV and STIs [25]. It may also be that settlement type is a proxy for sexual networks; studies in North America have highlighted how dense sexual networks, shaped by poverty and racism, are crucial for HIV-transmission patterns [26]. Finally, it may also be that people acquiring HIV are transitioning into urban informal settlements as they lose access to work and formal housing, and this explains the high HIV-prevalence. We could not, however, find a consistent measure of mobility across surveys to include this as a potential cofounder.

Our analysis also highlighted two trends in HIV-prevalence by settlement type. First, increasing HIV-prevalence amongst older (24–49) women in urban informal, rural informal and rural formal, and men (15–49) in rural informal. These changes may be indicative of the rollout of ARVs, with people surviving for longer, and HIV becoming a chronic disease [27]. The changing distribution of HIV also suggests that there may be important migration patterns as people age, with movement from urban to rural areas. The decreasing HIV-prevalence amongst younger (15–24) women in urban formal and urban informal settlements is likely indicative of the impact of improved prevention of mother-to-child transmission in South Africa [28], and also potentially the impact of HIV-prevention programming, reducing HIV-incidence [16]. The lack of similar trends in rural formal and informal settings highlights important gaps in impact for both programmes.

For men in 2012 the age stratified adjusted analysis showed that among young men those residing in rural formal areas had significantly higher HIV-prevalence than urban formal. Given that men typically acquire HIV at a later age [29], it may be the failure of prevention of mother-to-child transmission interventions in rural formal areas that leads to this patterning, paralleling what is seen among young women. For older men the sustained high HIV-prevalence in urban informal settlements, similarly parallels that seen among women of all ages, and is likely due to the rollout of ART. Importantly, given that one important pathway for HIV-transmission is often intergenerational [29], the high HIV-prevalence in older men in urban informal settlements could explain the continued high HIV-prevalence among young women in urban informal settings.

### Limitations

This study has a number of limitations. In all surveys, there was relatively high refusal rates for the main survey and for HIV-testing, particularly amongst men, and while data were weighted to account for this, there may be bias. In addition, the low levels of HIV-testing prevented age-disaggregated analysis of the men’s data, apart from for the 2012 data, and even in 2012 the large confidence intervals in adjusted analyses highlight the lack of precision. South Africa has high levels of mobility and internal migration, and the assumption that all participants were regular residents of the settlement type where data were collected cannot be tested. Similarly, there was variation in the timing of studies, and seasonal migration may have played a greater, or lesser role in depending on the months of data collection. In addition, only a few potential cofounders were consistently assessed in all four surveys, and key risk factors such as intimate partner violence, condom use, and migration were not included. We were unable to include the 2017 survey data as the data set had not been made public, and the categorization of settlement type was changed, with urban formal and urban informal combined into one category, ‘urban’, limiting comparability across time. Finally, only HIV-prevalence rather than incidence was assessed, and as such, we cannot determine whether the pattern of HIV-incidence is the same as prevalence. Nonetheless, analysis is nationally generalizable, as the surveys were all population based samples, and analyses were weighted to census data and to account for response refusal.

## Conclusions

The analyses presented have a number of implications for programming in South Africa. First, in terms of ensuring ARV coverage, and achieving 90-90-90, settlement types with the highest, and increasing HIV-prevalence (urban informal settlements, and rural informal and formal settlements) remain most poorly served. The historical legacies in South Africa remain around access to healthcare, despite attempts to address these via government. Spatially targeted access to ARVs is therefore an important priority. Second, given the concentration of urban informal settlements, in relatively geographically bounded, and dense situations, a significant effort is required around HIV-prevention programming. Current programmes such as DREAMS and She-Conquers, do recognize and target urban informal settlements [19], however, the specific challenges of interventions in these settings, and lack of well evaluated interventions delivered here, remains a major challenge to intervention programming.

More widely, this analysis suggests that settlement type may be important in understanding the spatial distribution of HIV in South Africa, and elsewhere. Such spatial patterning of HIV reflects patterns of wider marginalization of those who live there, including race, gender and poverty [1, 25, 26]. Further investigation into how these factors intersect to shape high HIV-incidence and prevalence is critical to understand how best to prevent HIV, and ensure HIV-treatment is available to all.
